# Retraction: Can artificial intelligence and face recognition using deep learning detect emotions in children with autism?

**DOI:** 10.1371/journal.pone.0340338

**Published:** 2025-12-26

**Authors:** The PLOS One Editors

Following the publication of this article [1] concerns were raised with the datasets used in this study. Specifically, the datasets reportedly present photographs of children with autism spectrum disorder. However, this diagnosis does not appear to have been validated. In addition, it is not clear whether the legal guardians of the children included in the dataset gave consent for these images to be distributed in a public dataset or used in academic research.

All authors responded stating that the use of the Kaggle datasets was a decision made in good faith, relying on what they considered to be the standard practice in the field of AI and Computer Science, and that they were unaware of the issues with the datasets.

In light of the concerns pertaining to the ethics and the validity of the dataset underlying the study, the *PLOS One* Editors retract this article [1]. PLOS regrets that these issues were not identified prior to publication.

This article [1] was removed from the *PLOS One* website on December 23, 2025. The article’s Copyright and Data Availability statements were updated at the time of retraction and removal, and the removed contents are no longer offered under the Creative Commons Attribution License.

All authors did not agree with the retraction.

## References

[1] Güngör İ, Sarman A, Tuncay S. RETRACTED: Can artificial intelligence and face recognition using deep learning detect emotions in children with autism?. PLoS One. 2025;20(12):e0338701. doi: 10.1371/journal.pone.0338701 41364738 PMC12688113

